# Photo-Cross-linked Gelatin Methacryloyl Hydrogels Enable the Growth of Primary Human Endometrial Stromal Cells and Epithelial Gland Organoids

**DOI:** 10.1021/acsami.4c08763

**Published:** 2024-07-18

**Authors:** Emma Salisbury, Thomas M. Rawlings, Spyridon Efstathiou, Maria Tryfonos, Komal Makwana, Harriet C. Fitzgerald, Caroline E. Gargett, Neil R. Cameron, David M. Haddleton, Jan J. Brosens, Ahmed M. Eissa

**Affiliations:** † Department of Chemistry, University of Warwick, Coventry CV4 7AL, U.K.; ‡ Division of Biomedical Sciences, Reproductive Health Unit, Clinical Science Research Laboratories, Warwick Medical School, University of Warwick and Tommy’s National Centre for Miscarriage Research, University Hospitals Coventry and Warwickshire NHS Trust, Coventry CV2 2DX, U.K.; § The Ritchie Centre, Hudson Institute of Medical Research, Clayton VIC 3168, Australia; ∥ Department of Obstetrics and Gynaecology, Monash University, Clayton VIC 3168, Australia; ⊥ Department of Materials Science and Engineering, Monash University, Clayton, Victoria 3800, Australia; # School of Engineering, University of Warwick, Coventry CV4 7AL, U.K.; ∇ Department of Polymers, Chemical Industries Research Division, National Research Centre, El Bohouth St. 33, Dokki, Cairo Giza 12622, Egypt; ○ School of Life Sciences, Faculty of Science and Engineering, University of Wolverhampton, Wolverhampton WV1 1LY, U.K.

**Keywords:** gelatin-based hydrogels, GelMA, 3D cell culture, primary human endometrial cells, endometrial organoids, *in vitro* tissue model, miscarriage

## Abstract

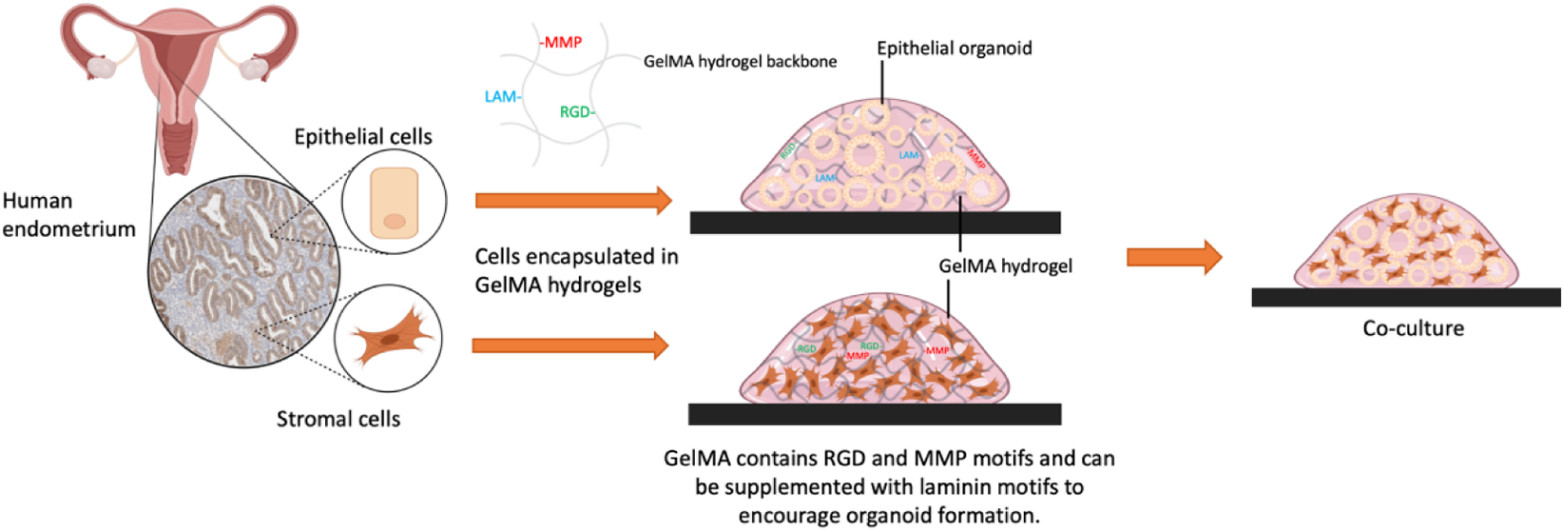

*In vitro* three-dimensional (3D) models are better able to replicate the complexity of real organs and tissues than 2D monolayer models. The human endometrium, the inner lining of the uterus, undergoes complex changes during the menstrual cycle and pregnancy. These changes occur in response to steroid hormone fluctuations and elicit crosstalk between the epithelial and stromal cell compartments, and dysregulations are associated with a variety of pregnancy disorders. Despite the importance of the endometrium in embryo implantation and pregnancy establishment, there is a lack of *in vitro* models that recapitulate tissue structure and function and as such a growing demand for extracellular matrix hydrogels that can support 3D cell culture. To be physiologically relevant, an *in vitro* model requires mechanical and biochemical cues that mimic those of the ECM found in the native tissue. We report a semisynthetic gelatin methacryloyl (GelMA) hydrogel that combines the bioactive properties of natural hydrogels with the tunability and reproducibility of synthetic materials. We then describe a simple protocol whereby cells can quickly be encapsulated in GelMA hydrogels. We investigate the suitability of GelMA hydrogel to support the development of an endometrial model by culturing the main endometrial cell types: stromal cells and epithelial cells. We also demonstrate how the mechanical and biochemical properties of GelMA hydrogels can be tailored to support the growth and maintenance of epithelial gland organoids that emerge upon 3D culturing of primary endometrial epithelial progenitor cells in a defined chemical medium. We furthermore demonstrate the ability of GelMA hydrogels to support the viability of stromal cells and their function measured by monitoring decidualization in response to steroid hormones. This study describes the first steps toward the development of a hydrogel matrix-based model that recapitulates the structure and function of the native endometrium and could support applications in understanding reproductive failure.

## Introduction

The human endometrium is a dynamic tissue that undergoes monthly cycles of shedding and regeneration in response to varying levels of the ovarian steroid hormones estrogen and progesterone. Following ovulation, a rise in progesterone levels triggers intensive remodeling of the endometrium, where multiple cell types work together to create the optimum environment for an implanting blastocyst.^1^ The endometrium consists primarily of epithelial glands surrounded by stroma. During the remodeling process, the endometrium differentiates, whereby the epithelial cells transform into a secretory phenotype and the stroma into specialized decidual cells.^2,3^ Proper functioning of the endometrium is essential for successful implantation and the continuation of pregnancy. Defects in endometrial function have been associated with a variety of reproductive disorders;^4^ however, due to a lack of *in vitro* endometrial models, our understanding of the cellular mechanisms surrounding these pathologies is incomplete.

*In vivo*, cells are surrounded by the extracellular matrix (ECM) which provides structural and biochemical support while regulating cell function. 2D cultures neglect to consider the complex effect of the ECM on the structure and function of cells. In 2D monolayer culture, cells often exhibit a flattened morphology and abnormal apical–basal polarity, this altered cell geometry and organization can directly impact cell function.^5^ Cells cultured in a monolayer will rapidly proliferate and spread, leading to cell senescence and loss of differentiated phenotype. By contrast, in a 3D environment, cell spreading occurs over a longer period of time and requires proteolytic cleavage of the physical scaffold.^6^ In order to model physiological events, there is a need for 3D culture systems that approximate the native environment *in vivo*. Biomaterial platforms have been explored as matrices to support the growth of cells in a 3D microenvironment and provide the necessary biophysical and biochemical cues for optimal cell–material interactions. Hydrogels allow cells to grow in a more physiological shape and can be engineered to better mimic the native environment of tissue; for example, their stiffness can be optimized to match different tissue types, and their surface functionality can be modified to promote cell adhesion and protein–protein interactions.^7,8^

Hydrogels can be classified into two distinct categories: natural and synthetic hydrogels.^9,10^ In cell culture applications, synthetic hydrogels often suffer from lower biological activity and do not facilitate cell interactions, acting as a passive platform for cells. Conversely, hydrogels derived from naturally occurring biopolymers are more commonly used due to their biocompatibility and low immunogenicity.^11−15^ Moreover, natural hydrogels possess components of the *in vivo* ECM and thus retain endogenous factors such as growth factors and bioactive motifs that promote cell adhesion and function,^16^ as well as allowing for cell-triggered remodeling.^17^

ECM-based hydrogel systems have been explored for the culture of endometrial cells, the most common being Matrigel and collagen.^18−20^ Matrigel is a commercialized product, consisting of a mixture of solubilized basement membrane proteins derived from mouse tumor cells, which at 37 °C form a physically cross-linked hydrogel. Matrigel hydrogels are widely used as a scaffold in 3D cell culture applications and are considered the gold standard for organoid culture protocols.^21^

Endometrial epithelial cells are a key component of the endometrium. These cells form the endometrial glands which secrete nutrients that act to nourish the developing embryo before the onset of intervillous placental perfusion around end of the first trimester.^22^ Turco et al. established endometrial gland organoids which can be grown from epithelial progenitor cells in a defined chemical medium.^23^ Organoids are self-organizing 3D-clustered cells, which retain key functional features of the tissue of origin, and routinely rely on use of Matrigel as the extracellular matrix.^24^ However, Matrigel hydrogels are ill-defined materials and can suffer from certain drawbacks, including high batch-to-batch variation in terms of composition, an undefined content, and lack of suitability for clinical applications due to being derived from tumors.^25^

Endometrial stromal cells are another major component of the endometrium and key to the establishment of a successful pregnancy. In response to rising progesterone and intracellular cyclic adenosine monophosphate (cAMP), stromal cells undergo a differentiation process termed decidualization, where they transform into specialized decidual cells which release secretory products such as prolactin.^26^ Decidual cells form a robust immune-privileged matrix preparing the uterus for pregnancy by permitting trophoblast invasion and vascular remodeling.^27^ Physically cross-linked collagen hydrogels have been shown to support the growth and differentiation of endometrial stromal cells;^19^ however, it has been reported that cell-induced contraction of the collagen matrix poses a major limitation for the utilization of such collagen hydrogels.^28^

It is worth mentioning that cell encapsulation in either Matrigel or collagen requires handling at low temperatures (usually in an ice bath) to avoid premature gelation, which can be experimentally challenging and deleterious for primary cells. More importantly, it is now becoming clear that the natural origin of both Matrigel and collagen limits their ability to mimic different and complex tissue environments.

To develop an *in vitro* model that can recapitulate the structure of the endometrium, biomaterials that support the growth and function of both epithelial and stromal cells are needed. Rawlings et al. reported an advanced endometrial model where they assembled stromal cells and epithelial organoids in a collagen hydrogel as assembloids.^29^ The assembloid model utilized a commercial type I collagen hydrogel; however, this is not an optimal matrix as it is not suitable for long-term culture due to its susceptibility to cell-induced breakdown and contraction. Use of the natural collagen hydrogel also offers no ability to tailor the matrix to better mimic the *in vivo* ECM. Using a more synthetic-based approach will allow for investigation into the optimal stiffness and biochemical motifs necessary for a superior endometrial model. Thus, there is a need to develop and evaluate new biomaterial tools that can support coculture experiments in order to provide a better understanding of endometrial tissue functions.

Gelatin-based hydrogels are attractive candidates for cell culture and tissue engineering applications. As a proteinaceous biopolymer obtained by partial hydrolysis of collagen (the major ECM component in most tissues), gelatin confers the hydrogel with arginine–glycine–aspartic acid (RGD) motifs that facilitate cell attachment as well as sites of matrix metalloproteinase (MMP) cleavage that allow for cell proliferation, migration, and remodeling.^30^ The use of a gelatin-based hydrogel precursor has a number of advantages over collagen including better solubility and lower antigenicity.^31^ Also, gelatin has reduced structural variations, compared to collagen, as hydrolysis process denatures the tertiary structure of collagen, and therefore, the properties of gelatin-based hydrogels are less dependent on fabrication parameters (e.g., source and gelation pH).^32^ At low temperatures (<30 °C), a gelatin solution gels and forms a physically cross-linked hydrogel. Modifying gelatin with methacrylamide and methacrylate groups leads to gelatin methacryloyl (GelMA) which can undergo photoinitiated radical polymerization upon exposure to appropriate UV light, forming covalently cross-linked hydrogels (referred to as GelMA hydrogel). GelMA hydrogels have improved mechanical properties over physically cross-linked hydrogels. Photo-cross-linking of GelMA enables tuning of the microfabrication and stiffness of the hydrogel to suit different applications, by varying the degree of substitution and/or concentration of the GelMA solution.^33^

These semisynthetic GelMA hydrogels have already been employed in various biomedical applications, including drug delivery, tissue engineering, and 3D culture of a wide range of cell types (e.g., neural stem cells,^34^ chondrocytes,^35^ mesenchymal stem cells,^36^ hepatocytes,^37^ and cancer cell lines,^38^ as well as in bioprinting to manufacture cell-laden constructs and microfluidic devices).^39^ Only a few preliminary studies suggested the suitability of GelMA hydrogels for the culture of endometrial cells.^40,41^ However, to the best of our knowledge, GelMA hydrogels have not yet been studied in detail for the culture of primary human endometrial cells or organoids, alone or in combination.

In this study, we report the fabrication of photo-cross-linked GelMA hydrogels with different mechanical properties achieved by altering two fabrication parameters: the degree of substitution and the concentration of GelMA. We then investigate these hydrogels as a platform for culturing primary human endometrial stromal cells (EnSCs) and epithelial glandular organoids. We determine an optimal matrix stiffness and explore modifying our GelMA hydrogels with the basement membrane protein laminin to create an enhanced matrix for epithelial organoid formation. All cell culture experiments were performed in comparison to previously reported protocols using commercial collagen/Matrigel hydrogels.^23,29^

## Methods

### Materials

Gelatin (type B, 225 bloom), methacrylic anhydride (MAA) 94%, lithium phenyl-2,4,6-trimethylbenzoylphosphinate (LAP), phosphate buffered saline (PBS) tablets, and ninhydrin were purchased from Sigma-Aldrich. Dialysis membranes (MWCO = 3.5 kDa) were purchased from Specta/Por.

Unless otherwise stated, reagents used for the culturing of primary endometrial cells were purchased from Life Technologies. The XTT cell viability kit was obtained from Cell Signaling Technology. Growth factor-reduced Matrigel was purchased from Corning Life Sciences, growth factor-reduced Geltrex was purchased from Gibco, and PureCol EZ Gel was purchased from Sigma-Aldrich.

### GelMA Synthesis

GelMA was synthesized following two different methods, A and B, leading to two different degrees of substitution (DS), DS70 and DS100, respectively.

#### Method A: For DS70 GelMA

GelMA was synthesized according to a previously described procedure.^42^ Briefly, 25 g of gelatin was dissolved in 250 mL of PBS at 50 °C. The pH was then adjusted to 7.4 using 4 M NaOH solution. 2.5 mL of methacrylic anhydride was added to the gelatin solution and left to react at 50 °C under continuous stirring for 3 h. The solution was then diluted with excess PBS before being transferred to 3.5 kDa dialysis tubing. The mixture was dialyzed against deionized water at 40 °C for 7 days to ensure complete removal of unreacted MAA and the methacrylic acid byproduct of the reaction. The resulting product was lyophilized and stored at 4 °C until further use.

#### Method B: For DS100 GelMA

Highly substituted GelMA was synthesized following a procedure reported by Zhu et al.^43^ Briefly, gelatin was dissolved in 0.25 M carbonate-bicarbonate buffer to 10% (w/v) at 55 °C. The pH of the solution was adjusted to 9.4 before 0.938 mL of methacrylic anhydride was added and left to react for 1 h at 55 °C, under stirring at 500 rpm. After 1 h, the pH was adjusted to 7.4 to stop the reaction. The final solution was dialyzed against deionized water at 50 °C for 7 days. The final product was lyophilized and stored at 4 °C until further use.

### Quantification of the Degree of Substitution (DS) of Gelatin

Methacrylation of gelatin was confirmed by using ^1^H NMR spectroscopy. Samples of 10 mg of gelatin and GelMA were dissolved in D_2_O for analysis. High-resolution ^1^H NMR spectra were recorded on a Bruker DPX-300 MHz instrument.

Infrared spectra were recorded using a Bruker ALPHA II Fourier transform infrared (FTIR) spectrometer.

The degree of substitution (DS) was defined as the percentage of free primary amine groups of gelatin that are replaced by methacrylamide groups in the resulting GelMA. It was determined by two methods: ^1^H NMR and a ninhydrin assay. The quantification of the DS using ^1^H NMR has been previously described.^44^ Briefly, the ^1^H NMR spectra were normalized to the phenylalanine signal (6.9–7.5 ppm), which remains constant throughout the reaction. The lysine methylene signals (2.8–2.95 ppm) from both the gelatin and GelMA spectra were integrated to obtain the areas. The DS of GelMA was calculated as follows:
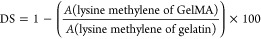
1

The ninhydrin assay was also used to determine the number of free amine groups on the lysine and hydroxylysine amino acid residues of unmodified gelatin and GelMA, following previously described methods.^45^ To generate a standard curve, a series of gelatin dilutions from 2 to 7.5 mg/mL were prepared. Ninhydrin was added to both gelatin standards and the modified GelMA sample at a final concentration of 2.7 mg/mL. Samples were incubated at 100 °C for 10 min. UV–vis spectra were recorded on an Agilent Technologies Cary 60 UV–vis spectrometer in the range of 450–700 nm for all samples. The absorbance max at 570 nm for each gelatin dilution was then plotted to form a standard curve (Figure S1).

For the modified GelMA sample, the fraction of free amine groups remaining was determined as follows:
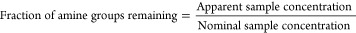
2

The nominal concentration refers to that at which the GelMA sample was prepared, while the apparent concentration was obtained from comparison with the gelatin standard curve.

The DS was calculated as follows:
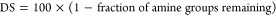
3

### GelMA Hydrogel Fabrication

GelMA hydrogels were prepared by photoinitiated radical cross-linking in the presence of the photoinitiator LAP. Four hydrogel formulations were prepared by dissolving GelMA in PBS: 5% DS70 GelMA, 10% DS70 GelMA, 5% DS100 GelMA, and 10% DS100 GelMA. GelMA solutions were incubated in a 37 °C water bath until complete dissolution of the GelMA solid and disappearance of foam. 0.1% w/v LAP was then added, and the resulting solution was cast into a 96-well plate. The plate was placed under 365 nm UV light for 3 min, resulting in the formation of hydrogel discs. The plate acted as a mold to create uniform hydrogel discs of 8 × 6 mm (diameter × height), which were used for rheological measurements and swelling assays.

### Swelling Behavior of GelMA Hydrogels

The swelling ratio of each GelMA hydrogel formulation was determined alongside collagen and basement membrane extract (BME: Matrigel or Geltrex) control hydrogels. The prepared hydrogels were transferred into a small beaker containing 30 mL of PBS and incubated for 3 h at 37 °C. The hydrogels were then weighed at appointed times (0.5, 1, 2, and 3 h). The degree of swelling of hydrogels at each time point was then calculated using the following formula:
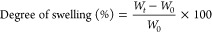
4where *W*_*t*_ refers to the weight at *t* min and *W*_0_ refers to the initial dry weight of the hydrogels at 0 min.

### Biodegradability of GelMA Hydrogels

The biodegradability of each GelMA hydrogel formulation was determined alongside collagen and BME control hydrogels. 5 μL of hydrogels was plated as droplets in a 96-well plate; droplets were cured under 365 nm UV light for 3 min and overlaid with stromal cell culture media. Droplet size was monitored and imaged using brightfield microscopy immediately postcuring and at the following time points: day 5, 10, and 15. Droplet size was measured in ImageJ.

### Rheology

The viscosity of GelMA hydrogels was determined by using an Anton Paar rheometer equipped with a parallel plate configuration (8 mm diameter). All amplitude and frequency sweep measurements were conducted at 37 °C. The storage modulus of each GelMA hydrogel sample was measured at a constant strain of 1%.

### Isolation and Maintenance of Primary Endometrial Cells

Endometrial biopsies were obtained from patients across 2 sites (Coventry and Monash). All participants provided written informed consent in accordance with the guidelines of the Declaration of Helsinki, 2000. For patients attending the Implantation Clinic, a dedicated research clinic at University Hospitals Coventry and Warwickshire (UHCW) NHS Trust, Coventry, UK, all biopsies were retrieved from the Arden Tissue Bank at UHCW. All research was undertaken with NHS National Research Ethics Committee approval (1997/5065). For patients at Monash Health, all human tissues were collected following ethical approval from the Monash Health and Monash University Human Research Ethics Committees (HREC).

Endometrial biopsies were collected in Dulbecco’s modified Eagle’s medium (DMEM)-F12 media that were supplemented with 10% dextran-coated charcoal (DCC)-stripped FBS and processed for primary culture as previously described, separating out human endometrial stromal and epithelial cells.^46^ Stromal cells were expanded in DMEM-F12 containing 10% DCC-FBS, 1% l-glutamine, 1% antibiotic–antimycotic solution, 1 nM β-estradiol (Sigma-Aldrich), and 2 μg/mL recombinant human insulin (Sigma-Aldrich). Stromal cells were used experimentally in passage 2.

Human endometrial epithelial cells (EpCs) were grown in hydrogel droplets straight after separation at passage 0. Hydrogel droplets were overlaid with an organoid expansion medium (Advanced DMEM/F12, N2 supplement, B27 supplement minus vitamin A, antibiotic–antimycotic solution, *N*-acetyl-l-cysteine (Sigma-Aldrich) 1.25 mM, l-glutamine 2 mM, recombinant human EGF (Peprotech) 50 ng/mL, recombinant human Noggin (Peprotech) 100 ng/mL, recombinant human R-Spondin-1 (Peprotech) 500 ng/mL, recombinant human FGF-10 (Peprotech) 100 ng/mL, recombinant human HGF (Peprotech) 50 ng/mL, A83-01 (Sigma-Aldrich) 500 nM, and nicotinamide (Sigma-Aldrich) 10 nM).

All cultures were maintained at 37 °C in a 5% CO_2_ humidified environment, and the culture medium was refreshed every 48 h.

### Preparation of GelMA Hydrogel Precursor Solution for Cell Culture

The required concentration of GelMA was dissolved in additive-free DMEM-F12, and solutions were vortexed thoroughly to ensure the GelMA was fully dissolved and left to rest in the 37 °C water bath until required. In preparation for cell culture, GelMA solutions were sterilized using a 0.2 μm syringe filter.

### Stromal Cell Culture

For stromal cell culture, cells were passaged at subconfluence by 5 min treatment with 0.25% trypsin-EDTA. The cells were pelleted and counted using a hemocytometer and trypan blue stain. The total number of cells required (50,000 cells/well) was added to an Eppendorf tube and centrifuged at 1200 rpm for 5 min. The media were carefully removed, and the cell pellet was resuspended in GelMA hydrogel precursor solution or ice-cold collagen. Samples mixed in collagen were kept on ice until plating, at which point the suspension was aliquoted in 20 μL droplets, one per well of a 48-well plate. Plates were incubated at 37 °C for 45 min before addition of media. For GelMA hydrogels, the LAP photoinitiator was added to the GelMA-cell suspension, and 20 μL of droplets was aliquoted to each well of a 48-well plate. The plate was cured under 365 nm UV light for 3 min. 200 μL of culture media was added to each well, and the medium was refreshed every 48 h. To induce differentiation, on day 5 of growth, EnSCs were downregulated in phenol-free DMEM/F12 media containing 2% DCC-FBS and decidualized with 10 μM of medroxyprogesterone acetate (MPA) and 0.5 mM of 8-bromo-cAMP.

### Epithelial Organoid Culture

For organoid culture, epithelial cells were grown and passaged as previously described.^47^ Upon separation from a biopsy, the cells were pelleted and gently resuspended in 20 × volume of ice-cold basement membrane extract (BME: Matrigel or Geltrex) or a GelMA hydrogel precursor solution. Samples mixed in BME were kept on ice until plating, at which point the suspension was aliquoted in 20 μL droplets, one per well of a 48-well plate. Plates were incubated at 37 °C for 15 min before addition of media. For GelMA hydrogels, the LAP photoinitiator was added to the GelMA-cell suspension, and 20 μL of droplets was aliquoted to each well of a 48-well plate. 250 μL of the expansion medium was added to each well, and the medium was refreshed every 48 h.

For passaging, BME droplets were collected into microcentrifuge tubes and centrifuged at 600 *g* for 6 min at 4 °C. Samples were resuspended in ice-cold, phenol red-free DMEM/F12 and then subjected to manual pipetting. Suspensions were centrifuged, resuspended in ice-cold additive-free DMEM-F12, and subjected to further manual pipetting. The suspensions were centrifuged again, and cell pellets were either resuspended in BME or GelMA hydrogel precursor solution and plated as described above.

### Assembloid Culture

Assembloids were established and decidualized as previously described.^29^ At passage 2, EnSC and gland-like organoid pellets were mixed at a ratio of 1:1 (v/v). The pellets were resuspended in either GelMA or ice-cold PureCol EZ Gel. The suspension was aliquoted and plated as described above, resulting in 20 μL volumes into each well of a 48-well plate. Expansion medium supplemented with 10 nM E2 was overlaid, and the medium was refreshed every 48 h. Decidualization was induced on day 4 of growth; assembloid cultures were switched to a minimal differentiation medium supplemented with 1 μM E2, 1 μM MPA, and 0.5 mM 8-bromo-cAMP for 4 days to allow for growth and expansion.

### Immunofluorescence Analyses

The medium was removed, and organoids were fixed in zinc formalin for 15 min. The organoids were washed in PBS and embedded in Tissue-Tek O.C.T. Compound, frozen at −80 °C, and sectioned at 7 μm. Sections were air-dried for 30 min and then postfixed in zinc formalin for 5 min and washed in PBS. Antigen retrieval was performed by incubating sections in boiling 1 × sodium citrate buffer, pH 6. After cooling for 30 min, sections were briefly washed in Milli-Q H_2_O, followed by PBST for 20 min and two washes of PBS (10 min each). The sections were blocked with 10% donkey serum (Sigma-Aldrich) for 30 min at room temperature, followed by overnight incubation with primary antibody at 4 °C. They were washed twice with PBS (10 min each) and incubated with either Alexa Fluor 488- or Alexa Fluor 568-conjugated secondary antibody (Thermo Fisher Scientific) in 2% donkey serum for 1 h at room temperature. The sections were then washed in PBS and incubated with Hoechst to visualize nuclei and imaged with a Olympus FV3000 Confocal laser scanning microscope.

### Enzyme-Linked Immunosorbent Assay

The spent medium was collected every 2 days during a 4-day decidual time course. Duoset solid-phase sandwich enzyme-linked immunosorbent assay (ELISA) kits (Bio-Techne) were used for the detection of PRL, OPN, and uPAR. Assays were performed according to the manufacturer’s instructions. Absorbance at 450 nm was measured on a PheraStar microplate reader with background subtraction from absorbance at 540 nm. Samples were interpolated from known standards by using a four-parameter logistic regression analysis.

### Hydrogel Digest and Analysis of Cell Viability

Enzyme solutions of collagenase I (Sigma-Aldrich) (1 mg/mL), collagenase V (1 mg/mL), dispase II (2.5 mg/mL), and trypsin/EDTA (2.5 mg/mL) were each prepared in PBS and incubated at 37 °C. Culture media were removed from wells. Hydrogels were washed once in PBS and then removed with a large bore pipet and placed in Eppendorf tubes. 200 μL of collagenase digest solution was added to each tube. Hydrogels were incubated in a 37 °C shaking water bath for 10 min. The tubes were centrifuged at 1200 rpm for 5 min to pellet cells, the supernatant was aspirated, and the pellet was resuspended in 200 μL of additive-free media. The number of live and dead cells was counted using trypan blue stain.

### Analysis of Cell Viability Using XTT Assay

Human endometrial stromal cell viability in GelMA hydrogels was measured using the XTT cell viability kit according to the manufacturer’s instructions. The assay detects cellular metabolic activities resulting in a color change that can be quantified by measuring absorbance. The working solution was prepared and added directly to the culture media on either cell monolayers or cell-laden GelMA hydrogels. After 4 h of incubation at 37 °C, the absorbance was measured at λ = 450 and 660 nm. Specific absorbance was used as a direct measure of the cell viability.

### Statistical Analysis

GraphPad Prism version 6 (GraphPad Software Inc.) was used for statistical analyses. *n* = 3 hydrogels per experimental group with the significance set as *p* = 0.05. Results are reported as the mean ± s.d.

## Results and Discussion

### Synthesis of DS70 and DS100 GelMA

**Figure 1:**
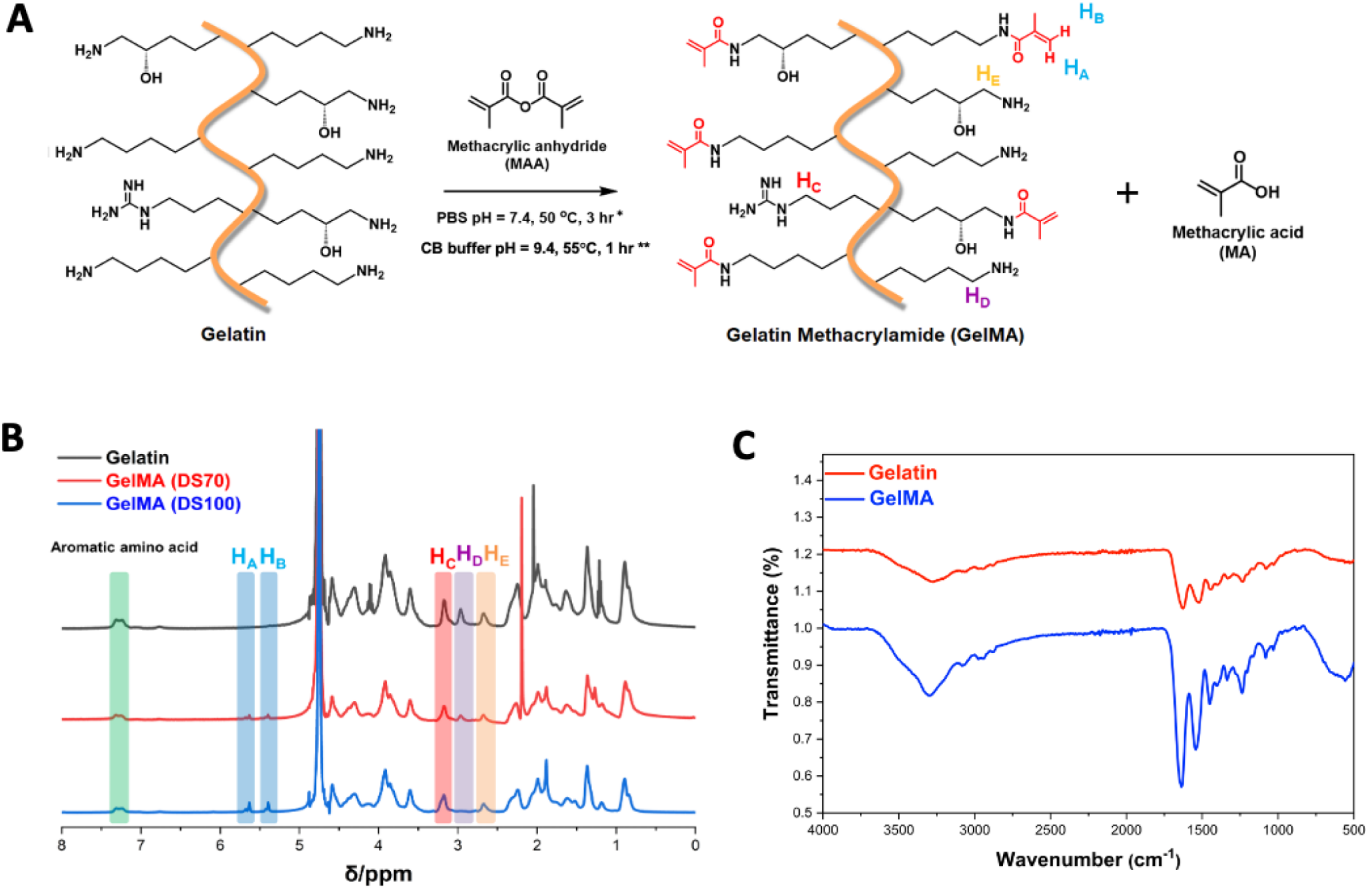
(A) GelMA synthesized from the reaction of gelatin with methacrylic anhydride (*reaction conditions for DS70 synthesis; **reaction conditions for DS100 synthesis). (B) ^1^H NMR spectra of gelatin compared to DS70 and DS100 GelMA, confirming the substitution of primary amine groups by methacryloyl groups in GelMA. Specific protons of gelatin and GelMA were highlighted as follows: acrylic protons of methacrylamide groups (H_A_, H_B_), methylene protons of arginine residues (H_C_), methylene protons of lysine residues (H_D_), and methylene protons of hydroxylysine residues (H_E_). (C) FTIR spectra of gelatin and GelMA.

GelMA is produced from the reaction of gelatin with methacrylic anhydride, where free amine groups contained within the side chains of the gelatin backbone are substituted with methacrylamide groups (Figure 1A). ^1^H NMR and FTIR spectroscopies were used to confirm successful substitution. The appearance of two new peaks (H_A_ and H_B_) observed at δ = 5.3 and 5.5 ppm in GelMA spectra was assigned to the acrylic protons of the methacrylamide groups (Figure 1B). FTIR spectra of gelatin and GelMA exhibit peaks at 3290 cm^–1^ associated with stretching of OH groups, and a strong peak appears in the GelMA spectra at 1640 cm^–1^ related to amide I, C=O stretching groups (Figure 1C).

The degree of substitution (DS) of GelMA can be altered by varying the ratio of MAA reacted with gelatin.^43,44,48^ GelMA with a target DS of 70% and 100% (DS70 and DS100) was synthesized to allow for the fabrication of hydrogels with a range of mechanical properties. The DS was calculated from the NMR spectra using eq 1, and the peak integrals of the methylene protons of lysine residues at 2.95 ppm (*H*_D_) were compared to the aromatic protons of phenylalanine residues at 7.3 ppm. The ratio of the peak integrals was found to be 5.4 for unmodified gelatin. For target DS70 GelMA, this ratio decreased to 1.39, suggesting a 74% substitution of the amine groups in the biopolymer product. For target DS100 GelMA, full substitution was confirmed by the complete disappearance of peak H_D_. To further confirm the DS, we used the ninhydrin assay to detect the presence of free amines.^45^ Using eq 3, we calculate a 65% substitution of the free amine groups in our target DS70 GelMA and a 97.5% substitution of the free amine groups in our target DS100 GelMA (Figure S1). These results are in good agreement with the DS values determined by ^1^H NMR and confirm the successful synthesis of both partially substituted GelMA (DS70) and fully substituted GelMA (DS100).

It is worth noting that gelatin has free amine groups present on the side chains of lysine, hydroxylysine, and arginine residues which have potential to react with methacrylic anhydride. However, amine groups on arginine side chains are not highly reactive, and methacrylic anhydride will predominantly react with lysine and hydroxylysine residues, leaving arginine residues unmodified.^19^ Crucially, for cell culture, this means active RGD (Arg–Gly–Asp) motifs are retained in the GelMA polymer, aiding cell attachment.

### GelMA Hydrogel Fabrication and Characterization: Swelling, Biodegradability, and Mechanical Properties

Upon UV light exposure, GelMA can rapidly undergo free radical photopolymerization in the presence of a photoinitiator to form cross-linked GelMA hydrogels. Irgacure 2959 is commonly used as a photoinitiator for cell encapsulation in hydrogels. In this study, we chose to use the photoinitiator LAP (lithium phenyl-2,4,6-trimethylbenzoylphosphinate) as this exhibits a higher solubility in water compared to Irgacure 2959, as well as a higher molar extinction coefficient at 365 nm, therefore enabling cell encapsulation at lower initiator concentrations and shorter UV exposure times.^49^ LAP is also cytocompatible and displays a reduced initiator toxicity, allowing for high levels of cell viability to be maintained during hydrogel encapsulation.^50^

GelMA hydrogels appear to form after as little as 1 min UV exposure time; however, the curing time used throughout this study was set at 3 min to ensure complete curing and minimize cell leaching from hydrogels. No running of GelMA droplets was seen upon tilting of the well plates after 3 min UV exposure (Figure S2). It was also observed that the higher the concentration of GelMA used, the quicker a cross-linked hydrogel formed upon exposure to UV, presumably due to the greater density of available cross-linking sites.

**Figure 2:**
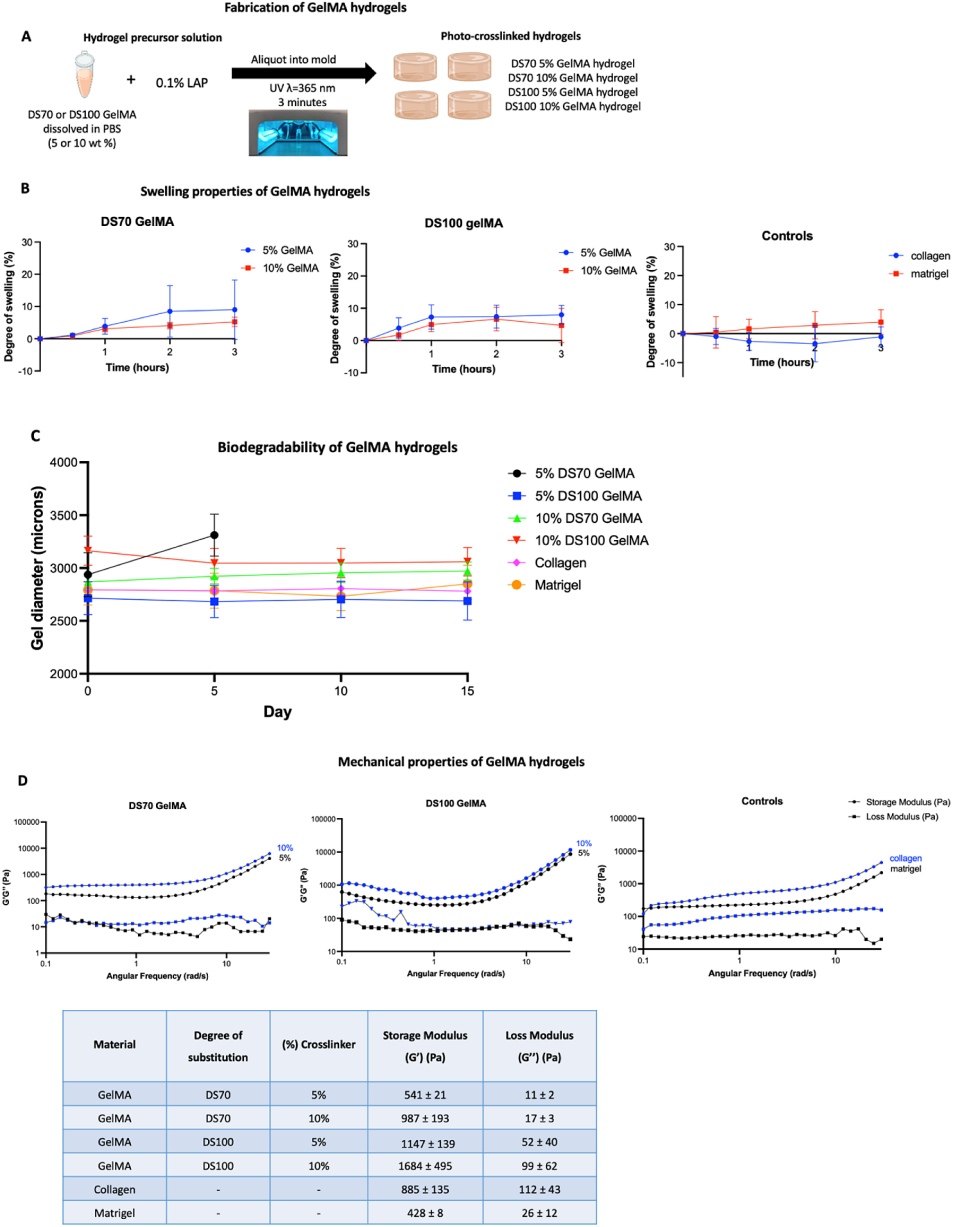
(A) Protocol followed for the fabrication of GelMA hydrogels. (B) Degree of swelling of DS70 and DS100 GelMA hydrogels of 5% and 10% (w/v), plus control collagen and Matrigel hydrogels, calculated at four different time points over the course of 3 h. (C) Biodegradability assay on GelMA hydrogels and control collagen and Matrigel hydrogel. (D) Rheological measurements of GelMA hydrogels and collagen and Matrigel hydrogels taken at 37 °C. *G*′ and *G*″ values were determined from frequency sweeps.

To study the effects of the DS and GelMA concentration on hydrogel swelling and mechanical properties, four hydrogel formulations were fabricated: 5% DS70, 5% DS100, 10% DS70, and 10% DS100 (Figure 2A). 5% was determined as the lowest weight percent possible to work with. Lowering the GelMA concentration further resulted in precluded curing of the hydrogel, even after extended periods of UV exposure (up to 30 min).

The degree of swelling was defined as the ratio of water taken up by the gel at a given time point during soaking, in relation to its initial dry weight. GelMA hydrogels were soaked in PBS at 37 °C. All hydrogels reached a swelling equilibrium within 2 h (Figure 2B). No degradation of the hydrogels was observed during the test; 10% GelMA hydrogels exhibited a lower swelling degree in comparison to 5% GelMA hydrogels. DS100 GelMA hydrogels also demonstrated a lower swelling degree in comparison to their DS70 counterparts with the same GelMA concentration. The higher the GelMA concentration, the greater the number of polymer chains and the higher the cross-linking density in the hydrogel; similarly, the higher the GelMA DS, the greater the number of methacryloyl cross-linking sites. Subsequently, the maximum volume increase of the gel is limited, resulting in less swelling.^51^ The highest degrees of swelling, up to 9.0 (±9.2) %, were seen in 5% DS70 GelMA hydrogels, reflecting the lower cross-linking density and increased capability for water uptake.

The biodegradability of a hydrogel is an important consideration for cell culture purposes; the hydrogel must remain intact for the length of the study. Hydrogels were left in the stromal cell medium (supplemented DMEM/F12) under cell culture conditions for a total period of 15 days. Hydrogel integrity was tracked using brightfield microscopy (Figure S3), and any changes in hydrogel size were quantified using ImageJ (Figure 2C). 5% DS70 GelMA hydrogels lost their structural integrity before day 5 in culture, with only 2 out of 6 hydrogels plated remaining, and these lost their shape, becoming very loose, and were easily removed with the media in the micropipette. We concluded that the 5% DS70 GelMA hydrogels are not suitable for cell culture purposes. All three other GelMA hydrogel formulations, as well as collagen and Matrigel retain their integrity over the entire 15-day timespan, with no significant change in hydrogel size observed.

The viscoelastic properties of the hydrogels were assessed through oscillatory rheology, by following the evolution of their storage (*G*′) and loss (*G*″) moduli. Frequency sweep experiments were performed at 37 °C to evaluate the effect of cross-linker concentration (5% and 10%) on the rheological behavior of the cured formulations in the angular frequency range of ω = 0.1–30.0 rad s^–1^. A constant strain of γ = 1.0% was selected based on amplitude sweeps conducted at a constant frequency of ω = 10 rad s^–1^. The formation of stable cross-linked networks was evidenced by the higher *G′* values compared to the *G*″ throughout the whole examined angular frequency range (Figure 2D). As expected, an increase in the concentration of GelMA led to a higher storage *G*′ value. Highly substituted DS100 GelMA hydrogels also displayed higher *G*′ values compared to lowly substituted DS70 GelMA hydrogels of the same concentration. More specifically, DS70 hydrogels containing 5% concentration of GelMA demonstrated the lowest *G*′ value, 541 ± 21 Pa, due to the lower number of cross-linking points. An increase in the cross-linking density was observed in DS100 hydrogels containing 5% GelMA leading to a *G*′ value of 1147 ± 139 Pa. DS100 GelMA hydrogels all demonstrated a higher *G*′ value compared to their DS70 counterparts, displaying a ∼ 2-fold increase in *G*′, suggesting a more rigid network due to the introduction of more cross-linking points. By altering the degree of substitution during the synthesis of GelMA and the concentration of GelMA used to form the hydrogels, we were able to tune the mechanical properties and create hydrogels with a range of strengths from 541 ± 21 to 1684 ± 495 Pa. We report a *G*′ value of 885 ± 135 for collagen hydrogels, placing them in the same strength range as our 10% DS70 GelMA hydrogels. Matrigel hydrogels exhibit a *G*′ value of 428 ± 8, indicating that they are the softest of all the hydrogels tested, with our closest strength GelMA hydrogel being 5% DS70.

Reported values for storage moduli of collagen and Matrigel vary greatly reflecting the significant batch-to-batch variation observed with natural hydrogels.^52−56^ We observed a high consistency in storage and loss moduli for each GelMA hydrogel, which will ensure consistency between experiments moving forward. GelMA hydrogel presents a physiological microenvironment with swelling and mechanical properties suitable for endometrial cell culture.

### Photoencapsulation of Primary Human Endometrial Stromal Cells (EnSCs) in GelMA Hydrogels

**Figure 3:**
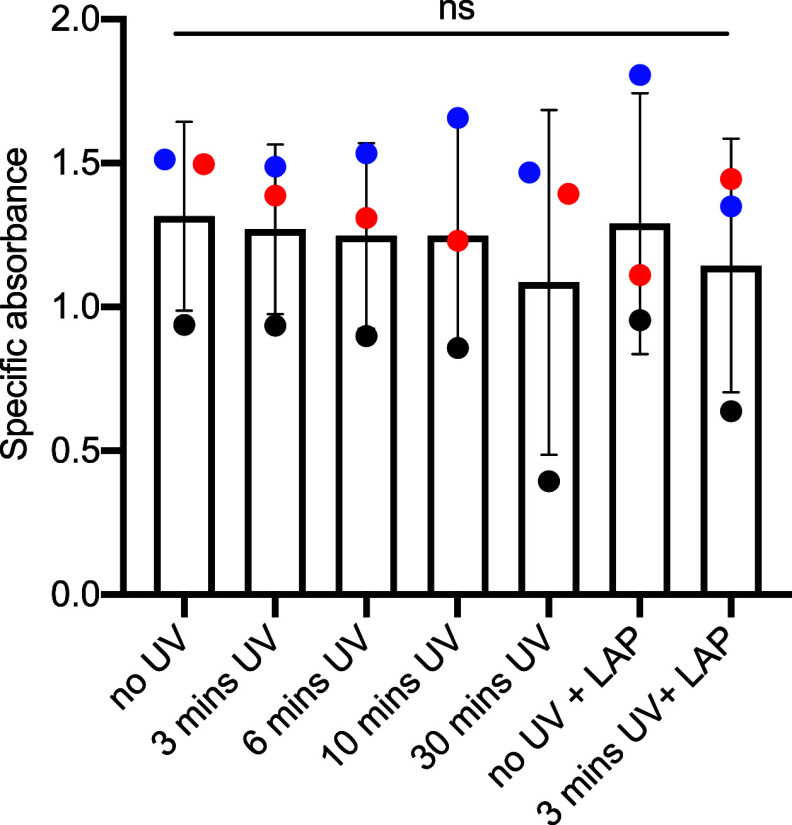
Viability of stromal cells under various conditions was determined by using the XTT assay. Exposure to 365 nm UV light was ranged over 0–30 min, and exposure to the LAP photoinitiator was tested. Each colored point represents a biological replicate (*n* = 3).

First, we wanted to confirm that neither UV exposure nor treatment with the photoinitiator resulted in a cytotoxic effect. EnSCs were exposed to UV light for various lengths of time ranging from 3 to 30 min. 48 h post UV exposure, cell viability was indirectly measured using the XTT assay where the specific absorbance correlated to cell viability.^57^ No change in cell viability was observed between nonirradiated control cells and those exposed to any of the different UV exposure times (Figure 3). The cytocompatibility of the photoinitiator was evaluated using both irradiated and nonirradiated LAP at a concentration of 0.1%. The cells displayed no change in cell viability compared to that of untreated cells. Based on these findings, the conditions of a 3 min curing time in the presence of the photoinitiator used for subsequent experiments throughout the study were deemed safe for cell culture experiments.

One challenge of culturing cells in hydrogels arises when cells need to be recovered for downstream molecular applications. The feasibility of recovering cells from GelMA hydrogels was tested by using an enzymatic digestion protocol. Four different enzymes were tested—trypsin, collagenase I, collagenase V, and dispase. These enzymes have demonstrated ability in digesting GelMA hydrogels; however, long digestion times, ranging from 40 min up to 8 h, have been required which is suboptimal for cell viability.^58,59^ Here, we were able to reduce the digest time down to just 10 min by adding in extra wash steps and mechanical aggregation. The viability of the cells recovered after the enzymatic digest was analyzed using the XTT assay (Figure S4). All of the enzymes tested maintained high levels of cell viability and cell recovery with averages of 83.15% (±12.0) and 67.97% (±16.69), respectively.

### Human Endometrial Organoid (HEO) Formation in GelMA Hydrogels

**Figure 4:**
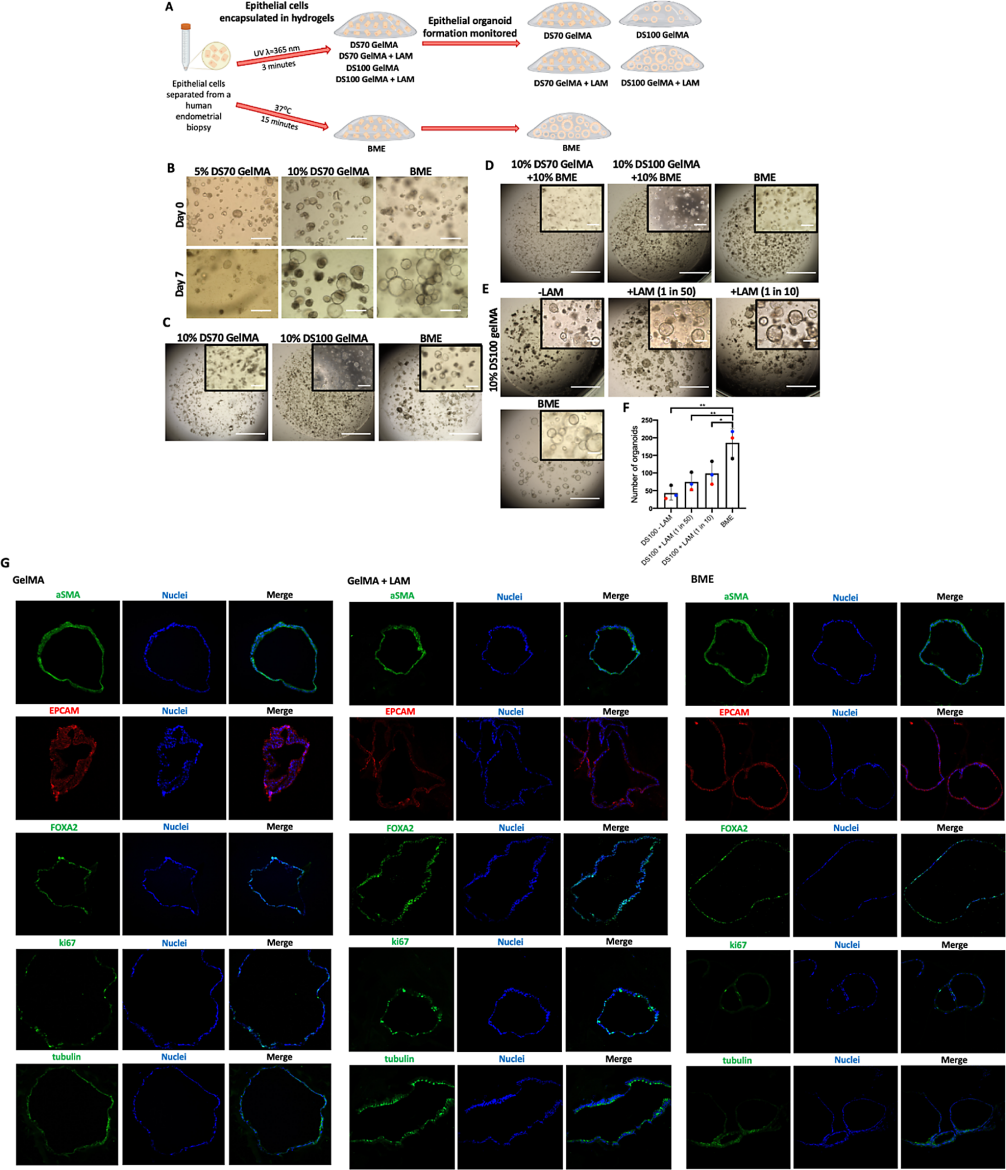
Organoid formation in GelMA hydrogels is supported by stiff matrix mechanical properties and enhanced by laminin supplementation. (A) Schematic overview of experimental design. (B) Representative images of endometrial organoids in stiff (10% DS100) and soft (10% DS70) GelMA hydrogels compared to those in BME. (C) Time-lapse images of organoids passaged into either GelMA or BME after a 14-day growth period in BME. (D) Day 6 images of organoids in GelMA–BME composite hydrogels. (E) Day 6 images of organoids in DS100 GelMA hydrogels supplemented with laminin protein compared to BME. (F) Quantification of organoid forming efficiency. Each colored point represents a biological replicate (*n* = 3). Scale bars = 400 μm. (G) Immunofluorescent localization of alpha-smooth muscle actin (aSMA), epithelial cell adhesion molecule (EpCAM), forkhead box A2 (FOXA2), ki67, and acetylated-α-tubulin (tubulin); in day 12, organoids grown in 10% DS100 GelMA and 10% DS100 GelMA + LAM or BME hydrogels. Nuclei were visualized with Hoechst stain.

The capability of GelMA hydrogels to support the formation and 3D culture of HEOs was examined in comparison to a BME (basement membrane extract) control. Initially, we looked at the maintenance and growth of organoid structures in GelMA hydrogels; organoids were formed and expanded in BME for 14 days and then passaged into 5% and 10% DS70 GelMA (Figure 4B). The organoids were imaged over a 7-day growth period; the higher concentration of 10% DS70 GelMA allowed organoids to grow and increase in size while retaining their morphology comparable to BME. However, 5% DS70 GelMA was not able to support organoid culture; the epithelial cells appeared to be unable to remain suspended in the hydrogel and instead grew in a 2D monolayer below the hydrogel. This highlighted how crucial the mechanical strength cues of the matrix are in the organoid culture. We next aimed to establish the optimal matrix stiffness for HEO formation.

Endometrial epithelial cells (EpCs) separated from an endometrial biopsy were directly encapsulated in 10% DS70 GelMA hydrogels and grown for 7 days; however, this matrix did not facilitate organoid formation (Figure 4C). To investigate if a stiffer GelMA matrix would allow for HEO formation, we synthesized a highly substituted DS100 GelMA with greater mechanical strength as demonstrated earlier. Interestingly, 10% DS100 GelMA demonstrated potential for organoid culture, and organoid structures were seen to be forming in the matrix albeit in smaller numbers compared to the BME control (Figure 4C).

To improve the efficiency of organoid formation, we explored the effect of basement membrane proteins by creating DS100 GelMA–BME composite hydrogels, thereby supplementing the cells with a mixture of basement membrane proteins. *In vivo*, epithelial cells interact with the basement membrane; importantly, this interaction provides the cells with survival, proliferation, and differentiation signals, as well as directional cues to establish polarity.^60,61^ As hypothesized, the presence of the basement membrane proteins allowed organoid formation in greater numbers in DS100 GelMA–BME similar to that seen in BME (Figure 4D). Nevertheless, the presence of basement membrane proteins was not able to rescue organoid formation in DS70 GelMA–BME.

Now, the cues needed for organoid formation had been determined: a stiff matrix and presence of basement membrane proteins; we wanted to refine the matrix. BME has an undefined composition, with one of the major components known to be the laminin protein. Laminin is present throughout the menstrual cycle in the glandular basement membrane.^62^ The laminin (LAM) protein was added into the GelMA hydrogels at two different ratios; the number of organoids formed in GelMA hydrogels increased with the LAM concentration (Figure 4E,F). Endometrial organoids formed in GelMA express the same marker proteins as those formed in BME hydrogels: EPCAM, a marker of columnar epithelium, and FOXA2, a marker of endometrial glandular epithelial cells, as well as acetylated tubulin, a marker of ciliated epithelial cells. Organoids in both GelMA and BME stain strongly for alpha-smooth muscle actin. Positive ki67 staining in GelMA hydrogels demonstrates cell proliferation; a higher number of proliferative cells were seen in comparison to BME suggesting that organoids formed in GelMA hydrogels have an extended proliferative growth phase compared to those formed in BME (Figure 4G).

Having already increased the DS to the maximum of 100%, we also tried increasing the concentration of GelMA from 10 to 15% to see if this could improve organoid forming efficiency further; however, organoids formed in smaller numbers when compared with 10% DS100. Therefore, we determined 10% DS100 GelMA as the optimal matrix strength for HEO formation (Figure S5).

### Human Endometrial Stromal Cell (EnSC) Culture in GelMA Hydrogels

**Figure 5:**
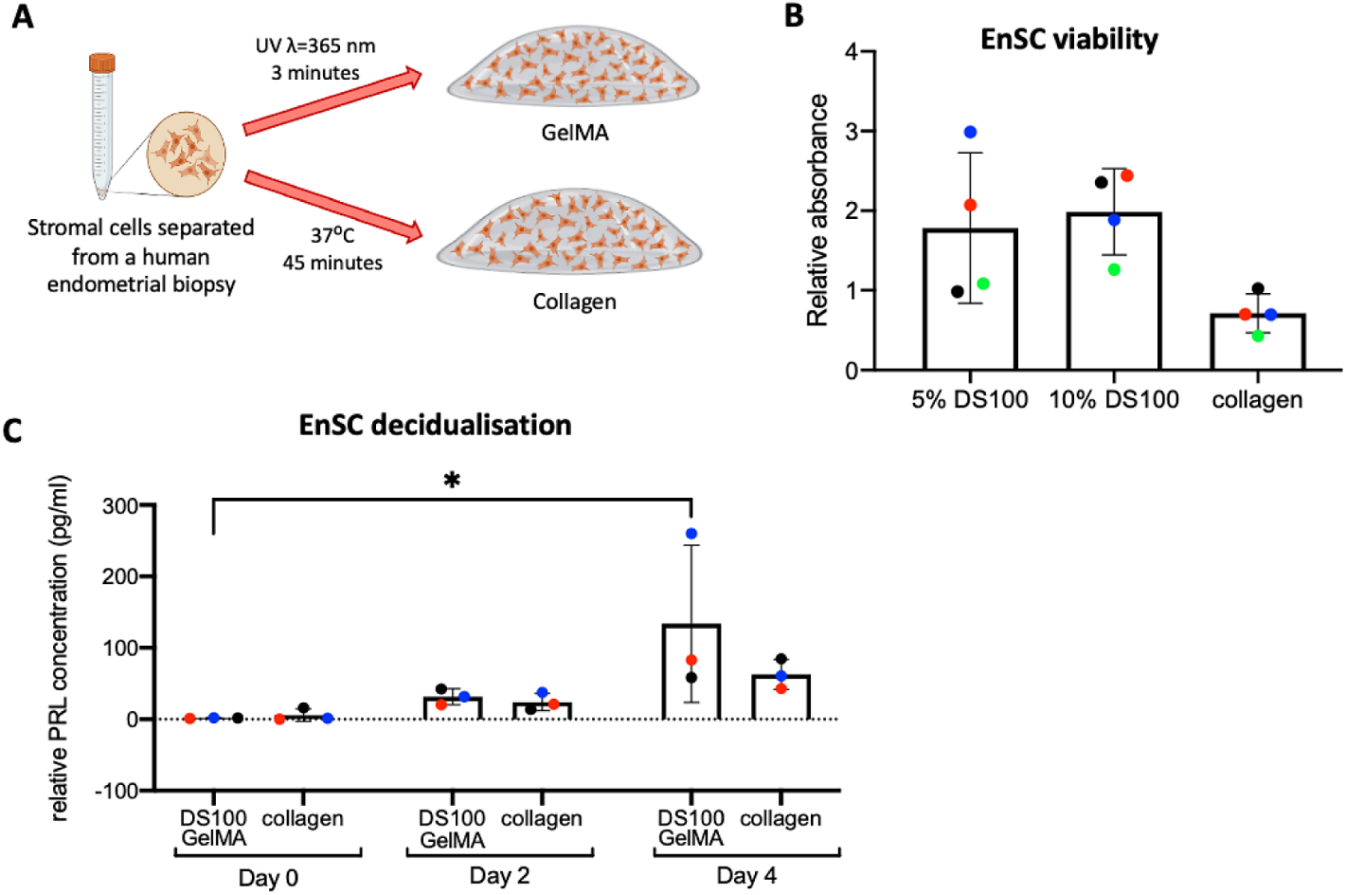
EnSC viability and differentiation are supported in DS100 GelMA hydrogels. (A) Schematic overview of experimental design. (B) XTT viability assay on EnSCs cultured for 5 days in 5% and 10% DS100 GelMA hydrogels compared to a collagen control. (C) ELISA quantification of prolactin secretion in DS100 GelMA compared to collagen. Each colored point represents a biological replicate (*n* = 3/4).

As DS100 GelMA was determined to be necessary for HEO formation, we continued working with this for stromal cell culture. Collagen was used as the benchmark for 3D stromal cell culture (Figure 5A). We examined the EnSC viability on day 5 of culture in both 5% and 10% DS100 GelMA (Figure 5B), where the XTT viability assay was employed. Both concentrations of GelMA supported a high level of cell viability and surpassed collagen cultures. To assess stromal cell function, 3D cultures were maintained for a further 4 days under steroid hormone treatment. Decidualization, a differentiation process EnSCs must undergo to accommodate pregnancy, was induced *in vitro* using a progestin (MPA) and a cyclic AMP analogue (8-bromo-cAMP) and characterized by the secretion of decidual marker prolactin (PRL).^3^ A clear differentiation response was seen in both GelMA and collagen hydrogels (Figure 5C). Time-lapse imaging was used to monitor EnSC morphology over the 9-day culture period (Figure S6).

### Future Outlooks

There is a strong demand for a system that can support the growth of multiple endometrial cell types. However, finding a suitable matrix has proven difficult; while Matrigel is optimal for organoid culture, it is not conducive to EnSC expansion. In contrast, collagen can support the growth of EnSCs yet does not support HEO formation.^50^ The use of collagen hydrogels is also associated with many limitations, including the unfeasibility of long-term culture due to gel contraction and degradation. More importantly, these traditional hydrogels only offer a fixed composition and mechanical strength, whereas GelMA hydrogels pose an alternative synthetic-based matrix that can be manipulated to better reflect the *in vivo* ECM and incorporate multiple endometrial cell types.

Following on from our success in HEO formation and EnSC culture in GelMA hydrogels, we will embark upon testing cocultures of stromal cells and epithelial organoids in our 10% DS100 GelMA + LAM hydrogels to examine cellular crosstalk. We have conducted a preliminary study where cell morphology was monitored over an 8-day culture period of 4 days growth followed by 4 days differentiation (Figure S7A). Significant contraction was seen in collagen hydrogels by day 8, while GelMA hydrogels maintained their structure and led to the formation of assembloids, similar to those previously reported by Brosens research group.^29^ Decidualization of assembloids with 8-bromo-cAMP and MPA resulted in robust secretion of gland-specific differentiation genes, uPAR and OPN, demonstrating the capability of gland organoids to respond to deciduogenic stimulation when grown in GelMA hydrogels (Figure 7B). Future work will examine cellular function in coculture and communication between the cell types.

## Conclusions

We described a GelMA hydrogel system that supports the culture of primary human endometrial stromal cells and gland organoids from endometrial epithelial progenitor cells. The protocol we have outlined for the simple fabrication of GelMA hydrogels requires exposure to UV light. The wavelength of UV light used to cure GelMA (λ = 365 nm) is very similar to that required for 3D printing purposes (λ = 405 nm), demonstrating the potential application in bioprinting. Gel formation was achieved quickly after one min exposure to UV light using a low-cost commercial UV nail polish curing lamp. Photo-cross-linked hydrogels were prepared with a range of mechanical strengths by altering the degree of substitution and concentration of GelMA. Hydrogels were fully characterized with their swelling and mechanical properties studied and determined as suitable for endometrial cell culture. Matrix mechanical stiffness was shown to be a key parameter in endometrial organoid formation. Stiff GelMA hydrogels (10% DS100) supported the formation and growth of HEOs, and formation efficiency could be enhanced with laminin supplementation. GelMA hydrogels supported the growth and differentiation of hormonally responsive EnSCs.

Unlike commercially available hydrogels such as collagen and Matrigel, GelMA hydrogels offer a tunable matrix; the mechanical properties can be altered to suit different cell types, and the material can be further functionalized to incorporate essential signals needed for different systems. Initial studies demonstrate potential for GelMA hydrogels to support coculture experiments and formation of endometrial assembloids and will be investigated further in the continued development of an *in vitro* endometrial model. We therefore foresee opportunities of opening new avenues for GelMA hydrogels in tissue engineering platforms to investigate the cellular processes that control human embryo implantation and implantation failure in a 3D environment.

## Supporting Information Available

Additional experimental details, including UV–vis spectra of standard gelatin solutions and its related standard curve, and figures showing images of experimental set up and brightfield images of organoid growth in GelMA hydrogels, as well as ELISA results of coculture experiments (PDF)
