# Supplementary material for: Photo-Cross-linked Gelatin Methacryloyl Hydrogels Enable the Growth of Primary Human Endometrial Stromal Cells and Epithelial Gland Organoids
Source: ACS Appl Mater Interfaces. 2024 Jul 18;16(30):39140–52. doi: 10.1021/acsami.4c08763 (PMC11299152; doi:10.1021/acsami.4c08763)
Supplement: Supplementary file 1 — am4c08763_si_001.pdf [file am4c08763_si_001.pdf]

Supporting Information

Photo-crosslinked Gelatin Methacryloyl Hydrogels Enable the Growth of Primary Human Endometrial Stromal Cells and Epithelial Gland Organoids

Emma Salisbury,[1] Thomas M. Rawlings,[2] Spyridon Efstathiou,[1] Maria Tryfonos,[2] Komal Makwana,[2] Harriet C. Fitzgerald,[3] Caroline E. Gargett,[3] Neil R. Cameron,[4,5] David M. Haddleton,[1] Jan J. Brosens[2] and Ahmed M. Eissa*[1,6,7]

[1] Department of Chemistry, University of Warwick, Coventry CV4 7AL, U.K.

[2] Division of Biomedical Sciences, Reproductive Health Unit, Clinical Science Research Laboratories, Warwick Medical School, University of Warwick and Tommy's National Centre for Miscarriage Research, University Hospitals Coventry and Warwickshire NHS Trust, Coventry CV2 2DX, U.K.

[3] The Ritchie Centre, Hudson Institute of Medical Research, Clayton, VIC 3168, Australia; Department of Obstetrics and Gynaecology, Monash University, Clayton, VIC 3168, Australia

[4] Department of Materials Science and Engineering, Monash University, Clayton, Victoria 3800, Australia

[5] School of Engineering, University of Warwick, Coventry CV4 7AL, U.K.

[6] Department of Polymers, Chemical Industries Research Division, National Research Centre, El Bohouth St. 33, Dokki, Giza 12622, Cairo, Egypt

[7] School of Life Sciences, Faculty of Science and Engineering, University of Wolverhampton, Wolverhampton, WV1 1LY, U.K.

E-mail: A.M.Eissa@wlv.ac.uk

A

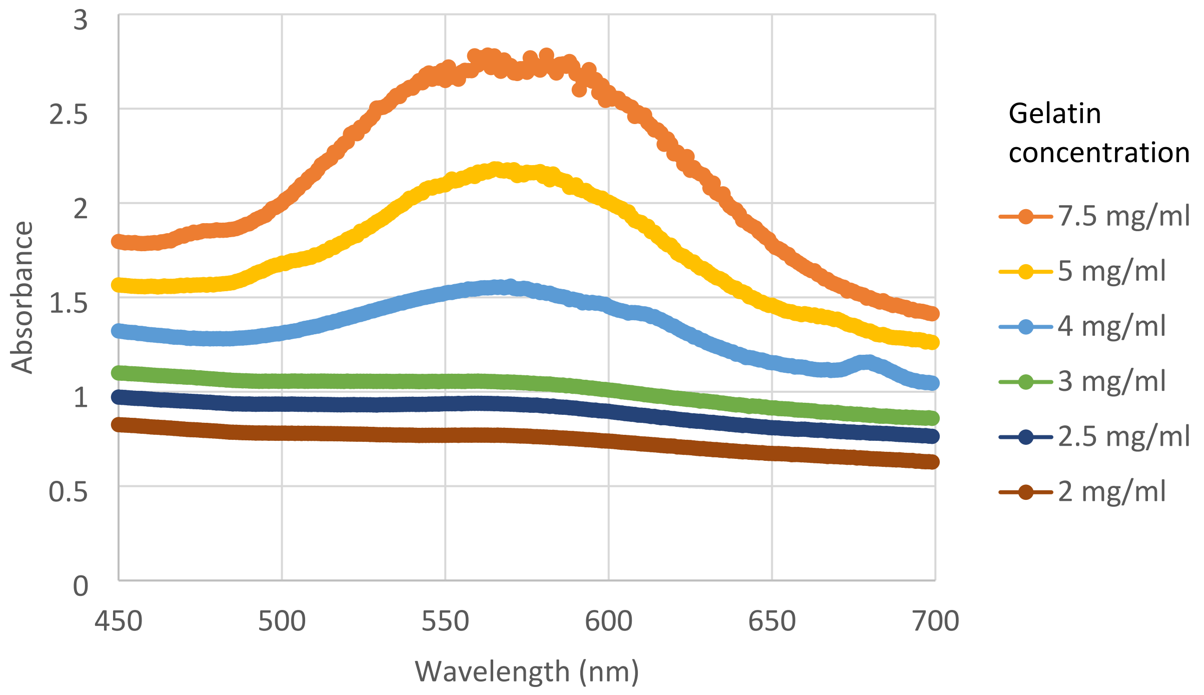

B

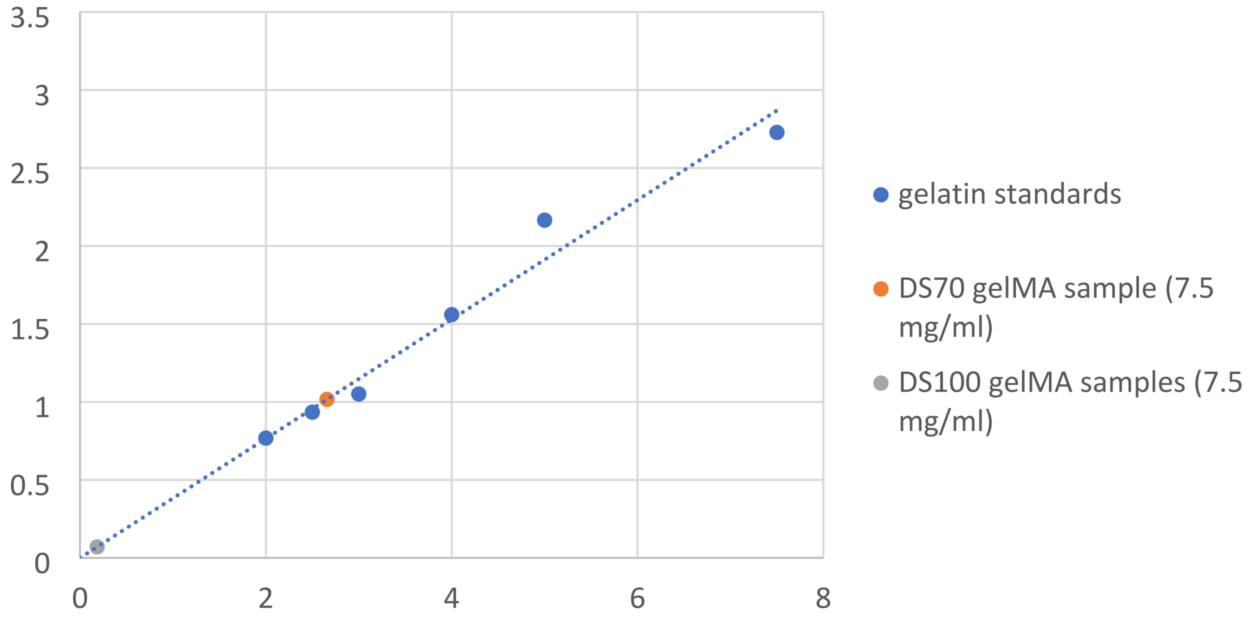

Figure S1. (A) UV-vis spectra of standard gelatin solutions (B) Standard curve showing absorbance intensity at 570nm of gelatin standards. Determination of DM for a representative sample of modified gelatin (orange circle). Here, DS70 gelMA prepared at 7.5 mg/mL exhibits an apparent concentration of 2.66 mg/mL (DM = 65%). DS100 gelMA prepared at 7.5 mg/mL exhibits an apparent concentration of 0.18 mg/mL (DM = 98%).

A

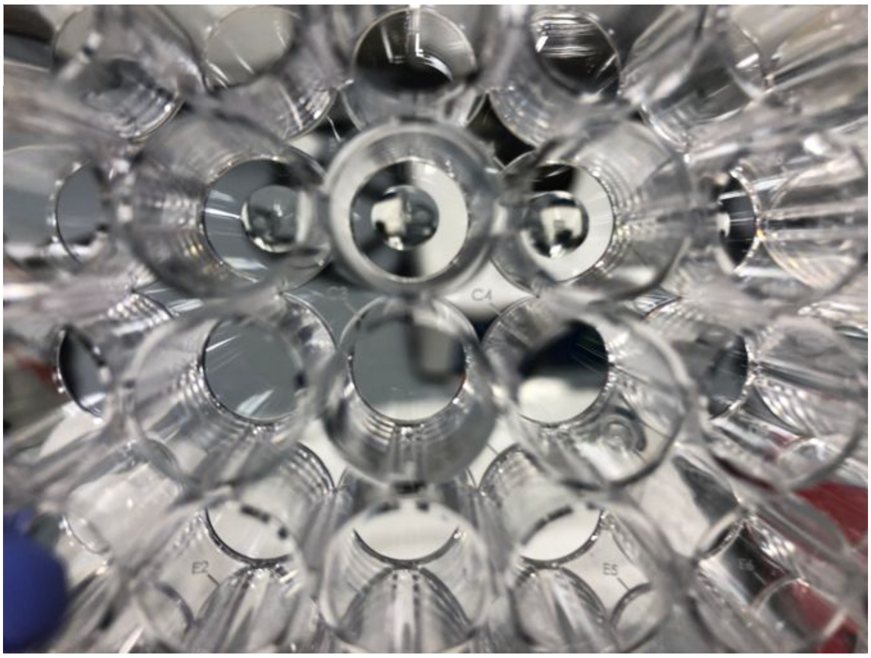

B

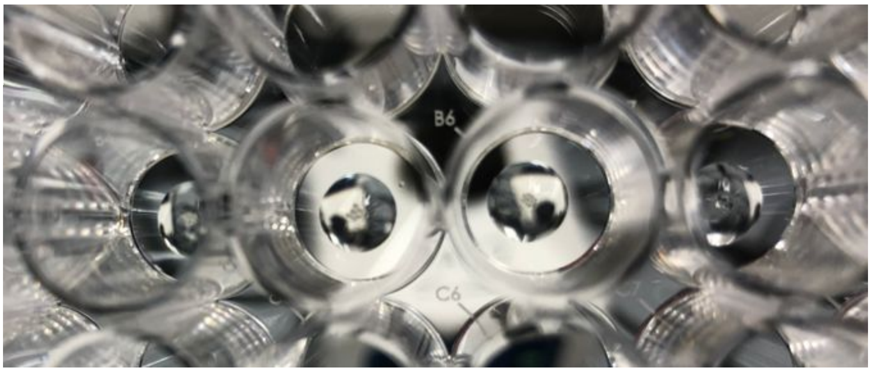

C

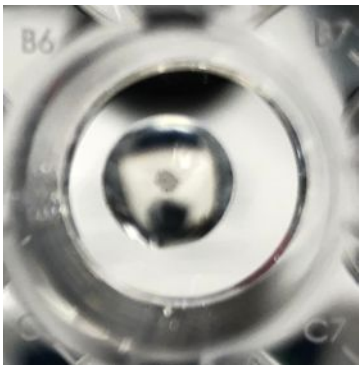

Figure S2. Images of gelMA droplets after 3 minutes UV curing (A) plate is being held up vertically to ensure droplets are fully cured and no running of the gel is visible (B) zoomed in view of row of wells (C) zoomed in view of an individual well.

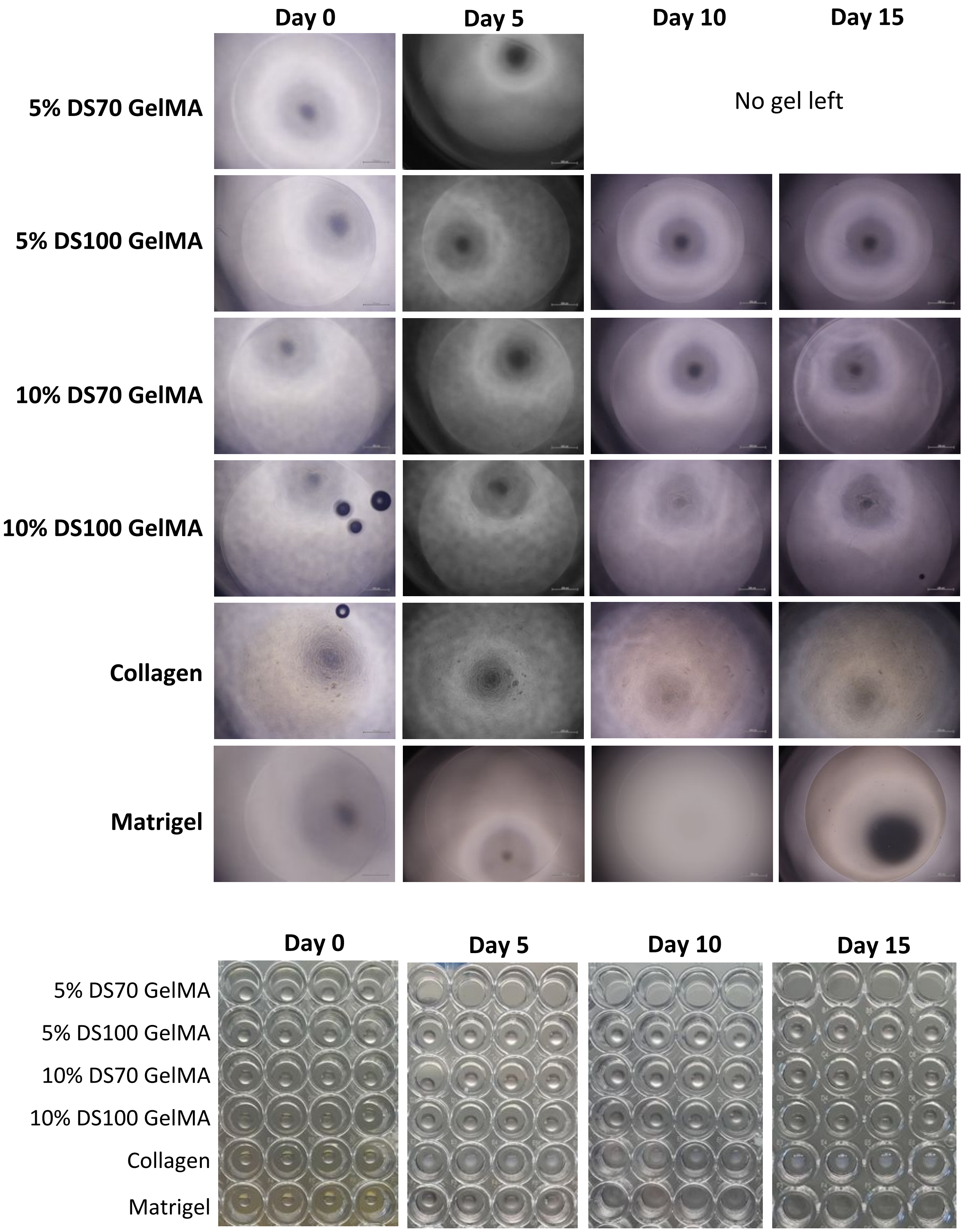

Figure S3. Images of hydrogel droplets over 15 days in culture.

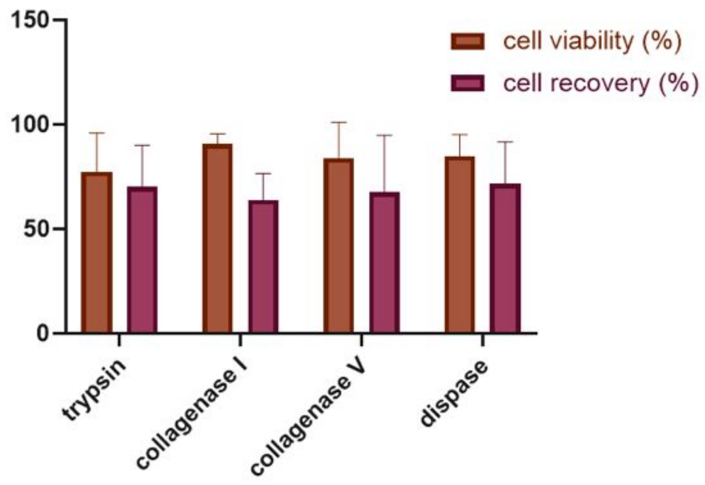

Figure S4. Viability and recovery of cells after a 10-minute digest with different enzymes.

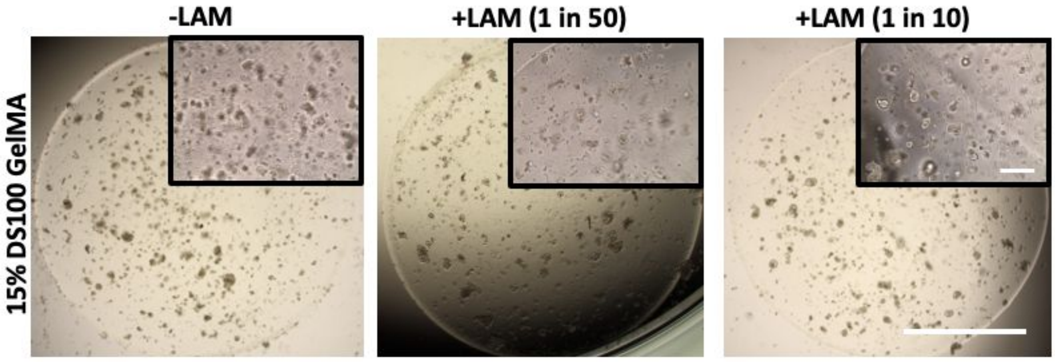

Figure S5. Brightfield images of organoid growth in 15% DS100 GelMA hydrogels supplemented with laminin protein. Scale bars = 400 μm.

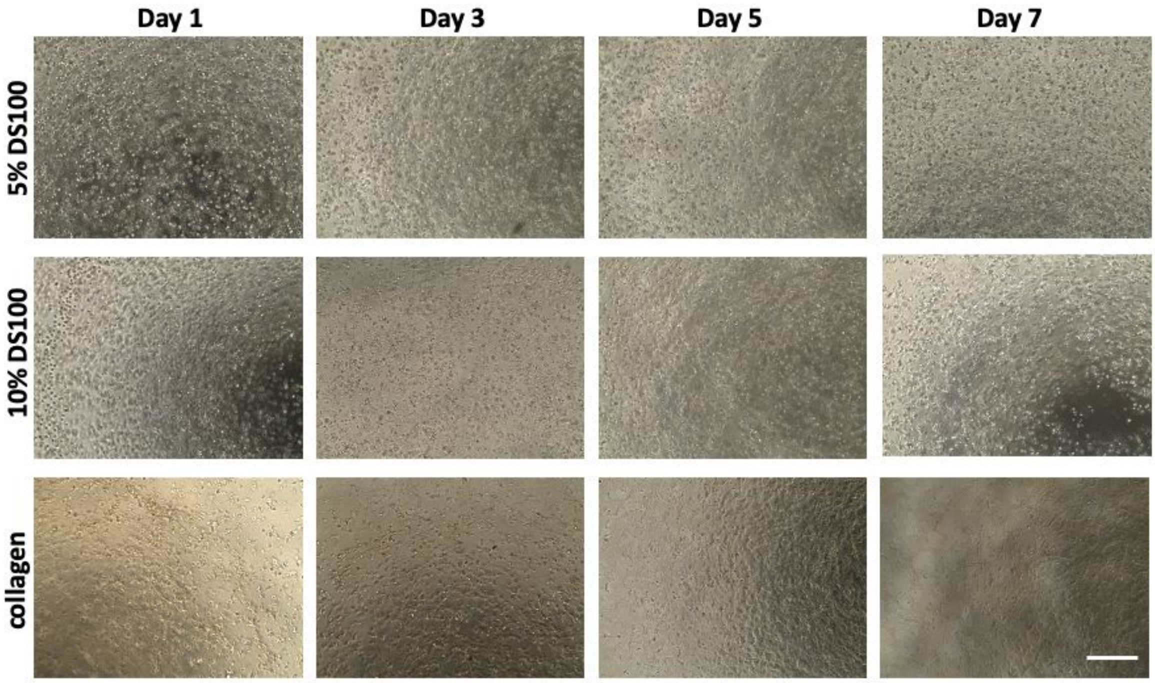

Figure S6. Brightfield images of stromal cells in GelMA hydrogels and a collagen control. Scale bar = 400 μm.

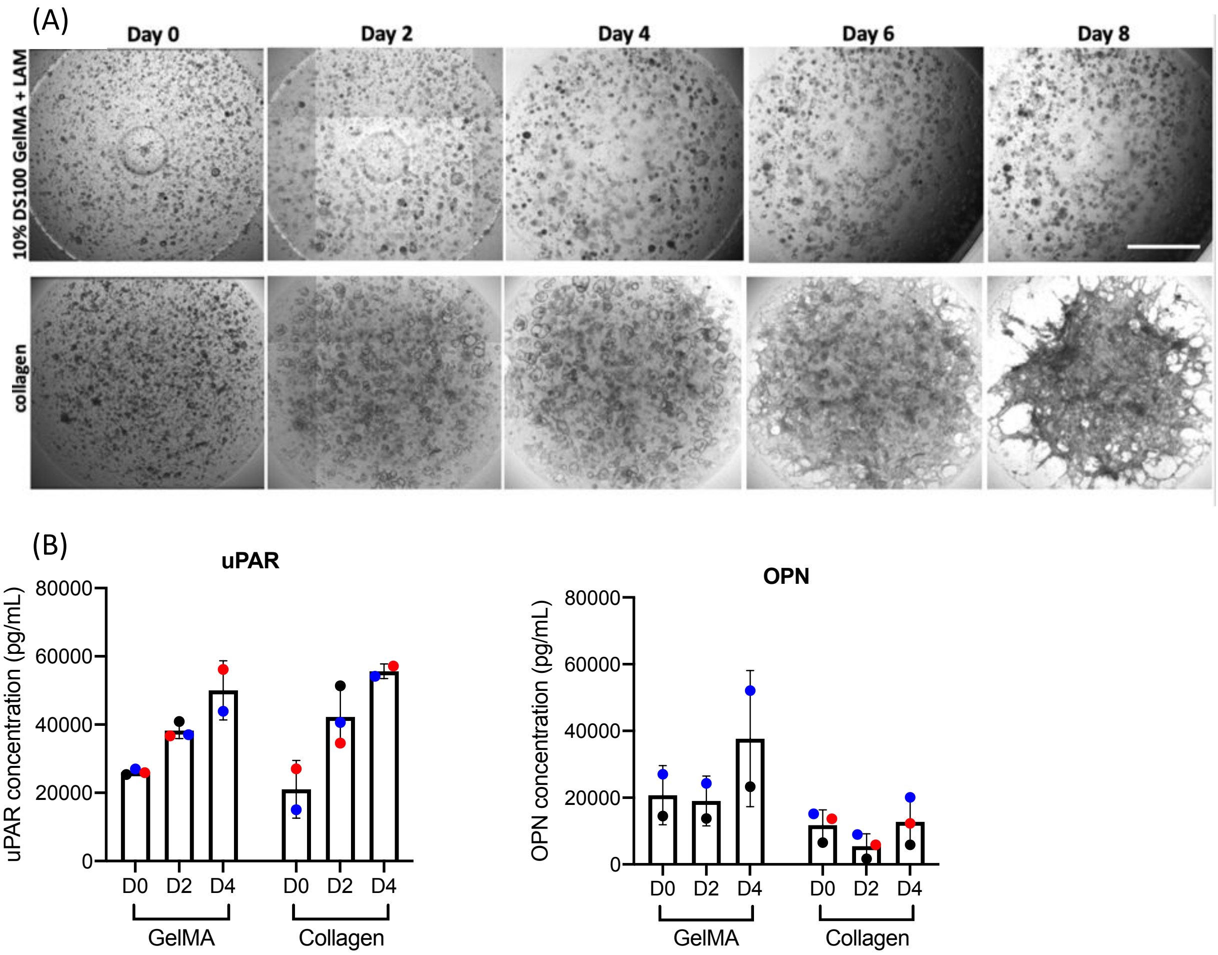

Figure S7. (A) Images of co-cultures over 8-day culture period. Scale bars = 400 μm. (B) Secreted levels of uPAR and OPN were measured by ELISA in spent medium at the indicated timepoints.
